# Virology and Immunology Researchers Evolve with the Viruses We Study

**DOI:** 10.1089/vim.2020.0232

**Published:** 2020-09-01

**Authors:** Rodney S. Russell

**Affiliations:** Division of BioMedical Sciences, Memorial University of Newfoundland, St. John's, Newfoundland and Labrador, Canada.

All species evolve to adapt to the environment in which they exist. As we sit in the middle of a global viral pandemic with our own environment changing every day at an uncomfortably rapid pace, one must wonder how much we are microevolving ourselves. Some changes are obvious, of course, the increased wearing of masks and the maintenance of physical distancing, but it will be years before we truly see how much this situation has affected us, and more importantly, our children. It is also clear and obvious that we are evolving, or at least adapting, on a professional level as scientists who are studying viruses and the immune system. A colleague recently mentioned to me that it must be an exciting time to be in this field, and it is. All fields of research evolve as new ideas and technologies emerge, but in the viral immunology world, a completely new disease can appear with the emergence of almost every new virus.

Anyone studying viruses knows that they can fall in and out of fashion quite quickly. The virus that is cool today might not be so cool tomorrow, and the hot new virus of this year might be gone by next year. Some virus researchers study a single virus for their whole career, whereas others study numerous viruses, sometimes unrelated, but I think all virology researchers at some point wonder whether they should switch to a different virus, or jump on the bandwagon and follow the money available to study the newest virus. What is clear is that our field is constantly changing, and most of us have to change with it because funding will always follow what is publically relevant. The resilience of immunology and virology researchers has always been apparent because the landscapes of our research fields have changed over the years. This notion is clear if we consider a few viruses such as HIV, HCV, and now SARS-CoV-2.

As with all viruses, when HIV was first discovered, understanding the virus and the immune response against it was the priority, but as we began to understand how the viral proteins functioned, and multiple classes of antiviral agents were discovered, we turned our attention to virus–host interactions and the identification of cellular factors that were critical for virus replication. Thirty years later, HIV infection is a chronic manageable disease in countries where therapy is available, but rightfully, the affected community said that a lifetime of treatment was not enough, and they asked for a cure. In response, funding priorities shifted, and many HIV researchers adapted and evolved, and we now have exciting efforts focused on finding new ways to target the latent pool of virus hiding in our own DNA. In this way, the status of HIV research forced immunology and virology researchers to rapidly adapt and evolve along with their field of research.

The evolution of HCV research took a similar path as that of HIV. We studied the virus, then the cellular factors, and the immune response for many years, but not everyone expected 99% cure rates to come along when they did, and that left many HCV researchers wondering whether studying this virus was still in their future. At that point, there was adaptation in many directions. Some stayed the course because there are indeed many important questions that still need to be answered regarding HCV, but some branched out conservatively, and others completely moved to different viruses. And as with HIV, the pursuit of an HCV vaccine remains to be a major priority, especially since we are still seeing more new infections annually than we are seeing individuals cured.

So here we are again in 2020. Our personal and professional environments have changed more in the past 6 months than they have in a 100 years, and all over the world immunologists and virologists, as well as scientists from numerous other fields, are joining the fight. Perhaps for some the decision is motivated by a need for research funding, but the result is still the same, we are adapting and evolving along with the field we work in. Some research programs are shifting focus completely, at least temporarily, while others are “growing new limbs” to incorporate coronavirus research. Hopefully we will not see another viral pandemic for at least another 100 years, but if we do, immunologists and virologists all over the world will be ready to adapt, evolve, and join the fight just as we have this time.

